# Progesterone Induces the Growth and Infiltration of Human Astrocytoma Cells Implanted in the Cerebral Cortex of the Rat

**DOI:** 10.1155/2014/393174

**Published:** 2014-05-22

**Authors:** Liliana Germán-Castelán, Joaquín Manjarrez-Marmolejo, Aliesha González-Arenas, María Genoveva González-Morán, Ignacio Camacho-Arroyo

**Affiliations:** ^1^Facultad de Química, Departamento de Biología, Universidad Nacional Autónoma de México, Ciudad Universitaria, Coyoacán, 04510 Ciudad de México, DF, Mexico; ^2^Laboratorio de Fisiología de la Formación Reticular, Unidad de Investigaciones Cerebrales, Instituto Nacional de Neurología y Neurocirugía MVS, 14269 Ciudad de México, DF, Mexico; ^3^Facultad de Ciencias, Laboratorio de Biología de la Reproducción animal, Departamento de Biología Comparada, Universidad Nacional Autónoma de México, Ciudad Universitaria, Coyoacán, 04510 Ciudad de México, DF, Mexico

## Abstract

Progesterone (P_4_) promotes cell proliferation in several types of cancer, including brain tumors such as astrocytomas, the most common and aggressive primary intracerebral neoplasm in humans. In this work, we studied the effects of P_4_ and its intracellular receptor antagonist, RU486, on growth and infiltration of U373 cells derived from a human astrocytoma grade III, implanted in the motor cortex of adult male rats, using two treatment schemes. In the first one, fifteen days after cells implantation, rats were daily subcutaneously treated with vehicle (propylene glycol, 160 **μ**L), P_4_ (1 mg), RU486 (5 mg), or P_4_ + RU486 (1 mg and 5 mg, resp.) for 21 days. In the second one, treatments started 8 weeks after cells implantation and lasted for 14 days. In both schemes we found that P_4_ significantly increased the tumor area as compared with the rest of the treatments, whereas RU486 blocked P_4_ effects. All rats treated with P_4_ showed tumor infiltration, while 28.6% and 42.9% of the animals treated with RU486 and P_4_ + RU486, respectively, presented it. Our data suggest that P_4_ promotes growth and migration of human astrocytoma cells implanted in the motor cortex of the rat through the interaction with its intracellular receptor.

## 1. Introduction

Astrocytomas are the most common and aggressive primary intracerebral tumors. They arise from astrocytes, glial progenitor cells, or cancer stem cells [1–5] and they are classified by the World Health Organization (WHO) in four grades (I–IV) according to their histological characteristics such as mitotic activity, nuclear atypia, cellularity, vascularity, and necrosis [6–8]. Anaplastic astrocytoma (WHO grade III) and glioblastoma (WHO grade IV) are the most frequent and malignant brain tumors in world population. They are characterized by high mitotic activity, nuclear atypia, and infiltrative lesions [9], and prognosis depends on multiple factors such as size, localization, and evolution time; however, generally, the survival of patients is very brief (24–36 months in anaplastic astrocytoma and less than 12 months in glioblastoma [10, 11]). Current medical treatments such as neurosurgery, radiotherapy, and chemotherapy achieve only a modest improvement in the length of survival and quality of life of patients [12–14].

Progesterone (P_4_) is a steroid hormone derived from cholesterol that regulates several functions such as sexual behavior, pregnancy, and neuroprotection, and it has also been related to cancer progression [15–17]. P_4_ exerts many of its effects through the interaction with its intracellular receptor (PR) which is a ligand-activated transcription factor [18, 19]. It has been reported that PR expression directly correlates with astrocytomas evolution grade, suggesting that PR-positive tumors present a high proliferative potential [20, 21].

It has been demonstrated that P_4_ promotes astrocytomas growth [22–25] and that the administration of RU486 (PR antagonist) blocks P_4_ effects [23, 26–28]. It has also been reported that RU486 improves the efficacy of chemoradiotherapy in glioblastoma xenografts in mice [29]. Previous studies about the role of P_4_ in astrocytoma cell lines proliferation* in vitro* have shown that P_4_ increases cell growth, as well as the expression of genes involved in cell cycle progression and metastasis such as cyclin D1, EGFR, and VEGF [30]; however, no* in vivo* studies have been performed. In this work, we studied the effects of P_4_ on tumor progression of U373 cells derived from a human astrocytoma grade III implanted in the motor cortex of the rat.

## 2. Materials and Methods

### 2.1. Cell Line and Culture

U373 astrocytoma cell line derived from a human astrocytoma grade III (ATCC, Manassas, VA) was maintained in Dulbecco's modification of Eagle's medium (DMEM) supplemented with 10% fetal bovine serum, 1 mM pyruvate, 2 mM glutamine, and 0.1 mM nonessential amino acids, all from Gibco (Grand Island, NY), at 37°C in a humidified atmosphere with 95% air/5% CO_2_. DMEM was changed every 48 hours until reaching 70–80% cellular confluence.

### 2.2. Implantation of Tumor Cells in the Rat Brain

The Wistar adult male rats (250–300 g) maintained on a 12 : 12 light/dark cycle with food and water ad libitum were intraperitoneally anesthetized with ketamine-xylazine (80/10 mg/kg resp.) and mounted in a stereotaxic apparatus. The head was cleaned and shaved, and the scalp was incised in the anteroposterior direction exposing the skull. Small holes were drilled in the left side of skull and a stainless-steel guide cannula (21-gauge) was inserted at the coordinates: Bregma AP = 1.6; *L* = 3.0, 2 mm above the injection site (motor cortex) according with the Paxinos and Watson atlas [31]. 120,000 U373 cells in a volume of 2 *μ*L of DMEM were slowly injected during a 2 min period using an injection cannula (25-gauge) inserted into the guide cannula connected through a polyethylene tube. The injection cannula that protruded 2 mm of guide cannula was maintained in the injection site for 5 more minutes after the injection. The hole bone was sealed using bone wax, and rats were given a dose of enrofloxacin (10 mg/kg) during 48 hours. All animal procedures were performed as per the following guidelines: (i) the Neurology and Neurosurgery National Institute's Ethical Code for the care and use of laboratory animals and (ii) Mexican guidelines for the production, care, and use of laboratory animals (NOM-062-ZOO-1999). The animals were maintained in the vivarium conditions until they were used.

### 2.3. Treatments

**Figure 1:**
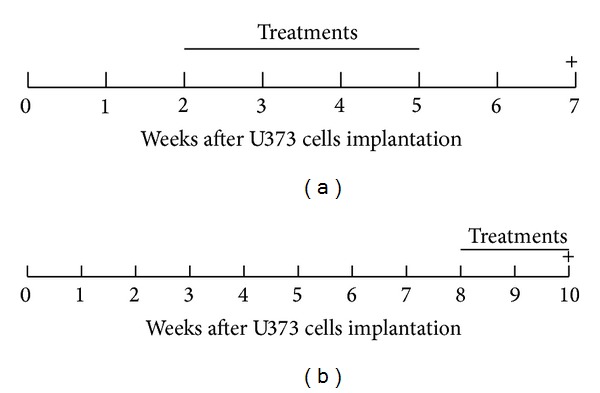
Scheme of treatments administered to the rats implanted with U373 cells in the motor cortex. (a) Short progression. (b) Long progression. + indicates the euthanasia.

Rats were randomly divided into four groups (7 rats/group), and each group was assigned to the following subcutaneous treatments (P_4_ and RU486 were dissolved in propylene glycol): vehicle (160 *μ*L of propylene glycol) (Baker Analyzed, Center Valley, PA); 1 mg of P_4_ (RBI, Natick, MA); 5 mg of RU486 (SIGMA, St. Louis, MO); or 1 mg of P_4_ + 5 mg of RU486. We performed two treatment schemes (Figure 1). In the first one (short progression), steroids were daily administered for 21 days, starting on day 15 after U373 cells implantation, and rats were euthanized 15 days after the last treatment. In the second scheme (long progression), we selected another 4 groups (2 rats/group) divided into the same way as described above, but the treatments started 8 weeks after U373 cells implantation; they lasted 14 days and the rats were euthanized one day after the last treatment.

### 2.4. Histology

Each rat was perfused with saline followed by 4% paraformaldehyde. Brains were removed and immersed in 4% paraformaldehyde at room temperature for 2 weeks. Afterwards, the brains were stored in sucrose gradient solutions (10%, 20%, and 30%) at room temperature for 24 hours each. Brain sections (10 *μ*m thick) were cut in the coronal plane around the implant site using a cryostat Leica CM1850 (Hesse, Germany). Some sections were stained by the Nissl method and examined in an Olympus Bx43 microscope (Tokyo, Japan).

### 2.5. Immunofluorescence

Another set of brain sections was blocked in 10% normal goat serum/0.05% Tween-PBS (blocking buffer) 1 hour at room temperature and incubated at 4°C overnight with primary antibodies that identified glioma and proliferating cells, respectively: mouse Anti SOX2 (1 : 50) (sc-365964, Santa Cruz Biotechnology, Dallas, TX) and rabbit Anti-Ki-67 (1 : 400) (Ab9260, Chemicon International, Temecula, CA) in blocking buffer. The antibodies were removed and the sections were washed three times with 0.05% Tween-TBS for 10 minutes and then incubated 1 hour at room temperature with secondary antibodies: Alexa 594 A-21203 (1 : 500) (Life Technologies, Carlsbad, CA) and FITC sc-2078 (1 : 500) (Santa Cruz Biotechnology, Dallas, TX). Nuclei were stained with Hoestch 33342 (Thermo Scientific, Waltham, MA). Sections were covered from light, washed, mounted with Fluoro Care Anti-Fade Mountant (Biocare Medical, Concord, CA), and visualized in an Olympus Bx43 fluorescence microscope. The tumor area and its infiltration length were quantified by using the program Image-Pro Plus 7.0 Media Cybernetics (Rockville, MD). The considered tumor area was the largest one of all the sections obtained from each brain, and the infiltration length was measured from the implant site to the longest distance reached by astrocytoma cells.

### 2.6. Statistical Analysis

Data from tumor area were analyzed by using ANOVA followed by the Bonferroni test for the comparison between groups. Infiltration length data were analyzed by using chi-square test. Prism 5.0 (GraphPad, San Diego, CA) was used for calculating probability values.

## 3. Results

**Figure 2:**
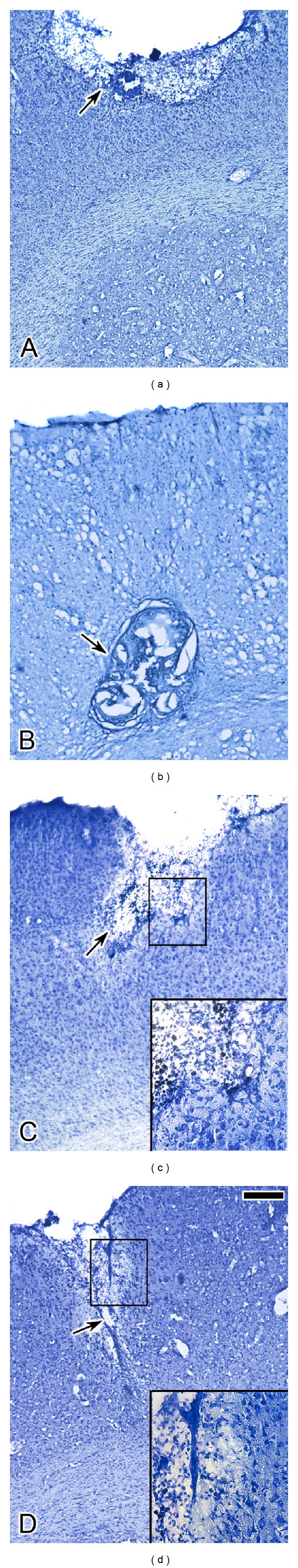
Effects of P_4_ and RU486 on the growth and infiltration of U373 human astrocytoma cells implanted in the motor cerebral cortex of the rat. Vehicle (propylene glycol) (a); P_4_ (b); RU486 (c); P_4_ + RU486 (d). Tumor cells are marked with an arrow. Magnification is represented by 200 *μ*m scale in (a)–(d) and by 100 *μ*m scale in the inserts (c)-(d).

In this work, we studied the effects of P_4_ and RU486 administration on the progression and infiltration of grade III human astrocytoma cells (U373) implanted in the motor cortex of the rat. In the Nissl stained brain sections from the short progression group, we observed that, in rats treated with vehicle, U373 cells stayed around the implantation area. In this group we did not find tumor infiltration (Figure 2(a)). In rats treated with P_4_, we observed both significant tumor growth and infiltration to deeper structures of the brain. The average distance covered by U373 cells with this treatment was 1119 ± 45.6 *μ*m (mean ± SEM). At the level of the corpus callosum, tissue structure was lost and we could not identify individual tumor cells; only amorphous structures were noticed (Figure 2(b)). Rats treated with RU486 (Figure 2(c)) showed a restricted tumor growth around the implant site with slight infiltration (82.2 ± 35.4 *μ*m). U373 cells were rounded or with elongated edges and they were smaller in comparison with normal astrocytic cells (insert Figure 2(c)).  Figure 2(d) shows a representative brain section of a rat treated with P_4_ + RU486. U373 cell morphology was diverse, showing variations in size and shape (insert Figure 2(d)). We observed that RU486 blocked P_4_ effects on tumor growth and invasion. Tumor infiltration (121.6 ± 43.3 *μ*m) was lower than that found in the rats treated with P_4_ and slightly higher (nonsignificant) than in the treatment with RU486.

**Figure 3:**
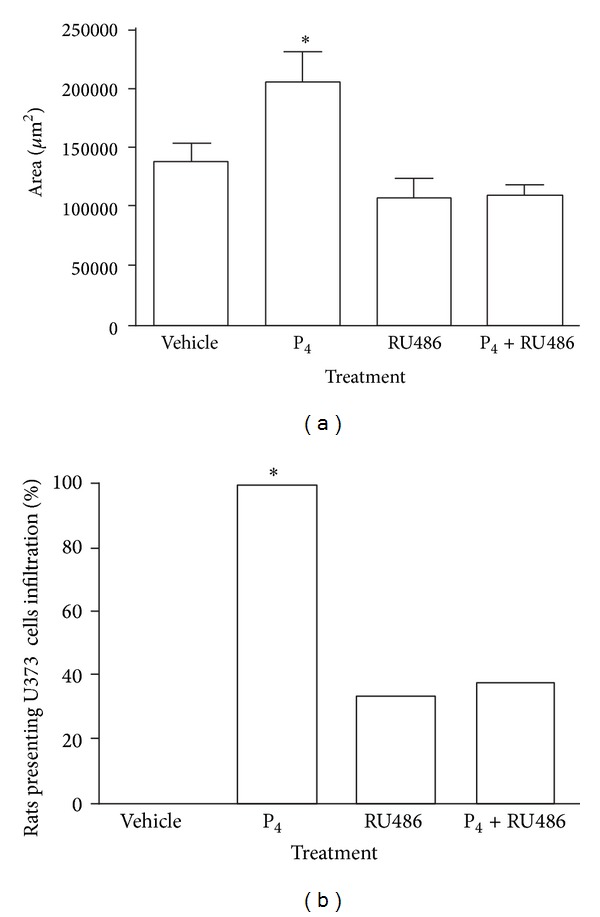
Effects of P_4_ and RU486 on the growth and infiltration of U373 cells implanted in the motor cortex of the rat. (a) Tumor area. Data represent mean ± SEM. (b) Percentage of rats with astrocytoma cells infiltration in the brain after treatments. *n* = 7. *P < 0.01 versus all groups.

P_4_ significantly increased both the tumor area of implanted U373 cells in the cerebral cortex of the rat (Figure 3(a)) and the infiltration length. Importantly, 100% of the rats treated with P_4_ showed cell migration toward deeper structures in the brain, while 28.6% and 42.9% of the animals treated with RU486 and P_4_ + RU486, respectively, showed it (Figure 3(b)). Although rats treated with vehicle presented a restricted tumor formation, they did not show tumor infiltration (Figure 3).

**Figure 4:**
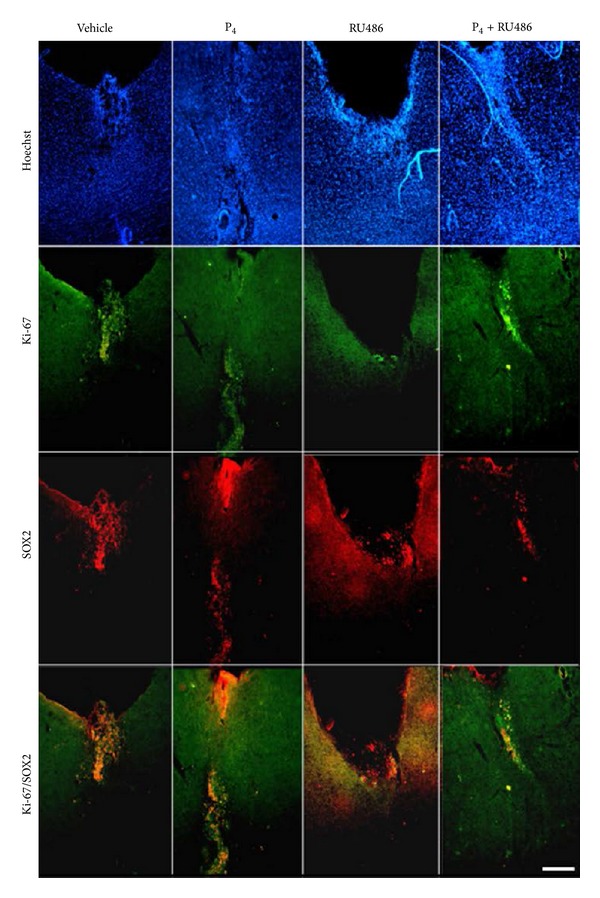
SOX2 and Ki-67 expression in U373 cells implanted in the motor cortex of rats under different treatments: vehicle, P_4_, RU486, or P_4_ + RU486. Each panel shows nuclei stained with Hoechst in blue, Ki-67 expression in bright green, SOX2 expression in red, and the colocalization of Ki-67 and SOX2 in orange. Magnification is represented by 100 *μ*m scale in all photomicrographs.

In the long progression group, despite the fact that we followed a different treatment scheme, the results of steroid administration were very similar to those of the short progression group (data not shown).  Figure 4 shows immunofluorescence staining of SOX2 and Ki-67 markers on brain sections of the long progression group treated with vehicle, P_4_, RU486, or P_4_ + RU486. As we observed in short progression group with brain sections stained with the Nissl method, in animals treated with vehicle, U373 cells stayed around the implant area, whereas, with P_4_ treatment, U373 cells migrated to deeper brain structures. In both treatments, Ki-67 and SOX2 were colocalized in 74% and 63% of the cells, respectively. Interestingly, we found that, in rats treated with RU486, there were just few cells positive to Ki-67 (18%) of the total cells that expressed SOX2, indicating the absence of proliferating glioma cells. Finally, in rats treated with both P_4_ and RU486, we noticed a decrease in U373 cells infiltration compared to those treated with P_4_, demonstrating that RU486 blocked P_4_ effects. With this treatment, Ki-67 and SOX2 presented colocalization in 48% of the cells.

## 4. Discussion

In the present study, we analyzed the effects of P_4_ and its antagonist RU486 on the growth and invasion of U373 cells implanted in the motor cortex of the rat. The increase in tumor growth after P_4_ administration observed in our* in vivo* conditions is consistent with the results observed in* in vitro* experiments with U373 cells [23, 26]. Additionally, it has been reported that, in U373 cells, P_4_ increases S-phase of the cell cycle [23] which could explain the increase in cell proliferation and therefore in tumor size. We also observed that RU486 blocked P_4_ effects, since rats treated with P_4_ + RU486 showed a significant decrease in tumor area in comparison with those treated with P_4_. These data are also consistent with previous reports in astrocytoma cell cultures [23, 26] and suggest that P_4_ effects on astrocytoma cell growth occur via the classic mechanism of action, through an interaction with PR.

P_4_ treatment also increased astrocytoma cells migration as well as the number of animals that presented tumor infiltration. These results have not been reported in brain tumors; however, there are studies in breast cancer indicating that P_4_ increases migration and invasion in MCF7 and T47D breast cancer cells and that RU486 treatment decreases migration [32]. It has been reported that progestins increase invasiveness in different cell lines of breast cancer. This effect can occur through various mechanisms, including overexpression of proteins such as superoxide dismutase, tissue factor, and protease-activated receptors [33–35]; the enhancing of matrix metalloproteinases and urokinase-type plasminogen activator activities [36]; the activating of the focal adhesion kinase [37], and the activation of rapid signaling cascades that leads to modifications in the actin cytoskeleton and the cell membrane [38, 39]. In other several cell lines, including glioma cells, it has been found that voltage-gated ion channels play a significant role in initiation and progression of cancer [40, 41] and even some of them as the potassium voltage-gated channel are regulated by P_4_ [42]. In our model, we observed that RU486 blocked P_4_ effects on tumor infiltration, suggesting that, as in the case of tumor growth, P_4_ effects occur through the interaction with PR; the precise mechanism involved in astrocytoma cells infiltration induced by P_4_ needs further investigation. Interestingly, although rats treated with vehicle presented a restricted growth of astrocytoma tumor area, no infiltration was observed in any rat unlike the animals treated with RU486 in which approximately 30% presented it (Figure 3). This may be due to a progestational action of RU486 that depends on the formation of specific RP dimers. Human PR presents two isoforms, PR-A and PR-B (94 and 114 kDa, resp.) with different function, regulation, and expression pattern. At basal state, PR is associated with heat shock proteins (HSP70 and HSP90) and once the hormone enters the cell, it interacts with PR and induces conformational changes that allow the dissociation of the HSP complex followed by phosphorylation and dimerization of the receptor. The active receptor possesses high affinity for specific sequences in the DNA called P_4_ response elements (PRE) that are found in the promoter region of P_4_ target genes. Once bound to PRE, PR can regulate gene transcription through the recruitment of corregulator proteins and the interaction with the basal transcription machinery [43, 44]. RU486 is a type II antagonist, which promotes PR dimerization and allows binding of the dimers to the PRE. It has been shown that RU486-bound A:A dimers are transcriptionally silent, whereas RU486-bound B:B dimers can activate transcription. RU486-bound A:B dimers act to distinctly inhibit transcriptional activation, and it is the activity that is commonly observed in P_4_ responsive cells [45, 46]. It is important to mention that PR-A and PR-B isoforms have been detected in human astrocytoma cell lines and biopsies, and their expression is directly related to the tumor evolution grade. Interestingly, PR-B content is three times higher than PR-A in U373 cells [23, 47] which could lead to an increased formation of B:B dimers and an activation of transcriptional activity upon RU486 treatment. However, the effects of this activation are significantly lower than those observed with P_4_ treatment. It has also been reported that, in astrocytoma tumors implanted in the cerebral cortex, the direction of migration is ventral through cortical gray matter and into the corpus callosum [48], which is consistent with our results.

Regarding the observed change in morphology of the implanted astrocytoma cells treated with RU486 (alone or in combination with P_4_), it has been reported that its administration induces alterations in the cellular structure of cancer cells of different origins (including glioblastoma cells). Such changes were associated with a redistribution of actin fibers that can form nonadhesive membrane ruffles, leading to a dysregulated cellular adhesion capacity and thereby altering the invasion capacity of these cells [49].

We observed that implanted cells expressed proliferation and glioma cells markers (Ki-67 and SOX2, resp.) and that, in many of them, both markers exhibited colocalization. These results demonstrated that the implanted U373 cells were present in the cerebral tissue of the rat and that they continued their proliferation. We also found that the percentage of Ki-67/SOX2 colocalization was higher in vehicle-treated rats that in those treated with P_4_. This could be related to the progression of these tumors induced by P_4_ leading to a dedifferentiation process where the resulting cells express less proliferation markers but overexpress invasion and/or migration markers. In the case of RU486 treatment, we observed very few cells positive to Ki-67 while those expressing SOX2 were found in a greater number. This indicates that there were glioma cells but they were not proliferating. It has been reported that RU486 induces G1-S blockage of the cell cycle in human ovarian cancer cells [50] and that RU486 reduces the activity of cdk2, enzyme that is involved in the regulation of the transcription factor E2F1 which modulates S-phase progression [51, 52].

## 5. Conclusions

P_4_ induces proliferation and infiltration of a tumor caused by the implant of human astrocytoma cells in the motor cortex of the rat through the interaction with intracellular PR.
